# Supplementary material for: Diet-wide analyses for risk of colorectal cancer: prospective study of 12,251 incident cases among 542,778 women in the UK
Source: Nat Commun. 2025 Jan 8;16:375. doi: 10.1038/s41467-024-55219-5 (PMC11711514; doi:10.1038/s41467-024-55219-5)
Supplement: Supplementary file 1 — Supplementary information [file 41467_2024_55219_MOESM1_ESM.docx]

**Contents**

[Supplementary Methods 2](#_Toc183081728)

[Assessment of dietary intakes using the dietary questionnaire 2](#_Toc183081729)

[Measurement error correction 4](#_Toc183081730)

[Supplementary Table 1. Relative risks and 95% confidence intervals of colorectal cancer per 300 mg/day higher intake of calcium based on calcium intakes from the baseline dietary survey and based on calcium intakes from the baseline dietary survey and re-measured calcium intakes from the Oxford WebQ combined 4](#_Toc183081731)

[Supplementary Figures 5](#_Toc183081732)

[Supplementary Figure 1. Participant flowchart 5](#_Toc183081733)

[References 6](#_Toc183081734)

# Supplementary Methods

## Assessment of dietary intakes using the dietary questionnaire

The dietary questionnaire (forming the baseline for this study) asked participants about their diet during a typical week, including 130 quantitative or semi-quantitative questions on frequency of intake of specific foods and food groups.

*Meat*

For the questions on meat, women were asked to select the types of meat they consumed about once a week or more from the following types: beef, bacon, chicken/poultry, lamb, ham, kidney, pork, sausages, liver/pâté, beefburger/hamburger. They were also asked if they never ate meat. A separate question asked women to report the total number of times meat was eaten per week. For these analyses, we determined the portion of different meat types consumed by combining the information on consumption frequency with the types of meat selected and multiplying these by standard portion sizes. We then summed intakes by groups as follows: chicken, red meat (beef, pork, lamb, kidney, liver, burger), and processed meat (sausage, bacon, ham). We also combined red and processed meat. Intake frequencies were grouped into fifths and we estimated trends in risk across these baseline categories.

*Fish and seafood*

For the questions on fish, women were asked to select the types of fish they consumed about once a week or more from the following types: tuna, sardines, trout, fish and chips, salmon, mackerel, kippers/herring, cod/haddock/other white fish, and other seafood. They were also asked if they never ate fish. A separate question asked women to report the total number of times fish was eaten per week. For these analyses, we determined the portion of different fish and seafood types consumed by combining the information on consumption frequency with the types of fish selected and multiplying these by standard portion sizes. We then summed intakes by groups as follows: oily fish (sardines, salmon, trout, kippers, and mackerel), and non-oily fish (seafood, fish and chips, tuna, and white fish). Participants were then divided into groups of intakes (<1, 1+ wk) and we estimated trends in risk across these baseline categories.

*Vegetables and legumes*

For the questions on vegetables and legumes, women were asked to select the types of vegetables and legumes they consumed about once a week or more from the following types: aubergine, carrots, courgettes, beetroot, parsnip, lettuce, tomatoes, swede, spinach, sweetcorn, avocado, celery, green beans, green/red peppers, mushrooms, cucumbers, broccoli, cabbage, cauliflower, brussel sprouts, onion, garlic, leeks, green peas, baked beans, chick peas/lentils, soya/tofu. Two separate questions asked women to report the total number of times cooked vegetables and salad/raw vegetables were eaten per week. To rank the women, we grouped the intake frequencies across these two questions and grouped them into the following categories: <10, 10-14, 15-19, 20-29, 30+ pw. We then estimated trends in risk across these baseline categories. We also included the 26 individual vegetable and legume items as they were asked (grouped as once or more per week versus less than once per week). Aubergine consumption was not asked about in the Oxford WebQ and was therefore excluded from this analysis.

*Fruit*

Women were asked to select the types of fruit they consumed about once a week or more from the following types: apples, bananas, oranges/satsumas, grapefruit, pears, stone fruit. We then categorized women into those who reported eating fruit less than once per week, and once or more per week. Three separate questions asked women to report the total number of times fresh fruit, dried fruit, and stewed or tinned fruit were eaten per week. For dried fruit, and stewed or tinned fruit we categorized women into those who reported eating fruit less than once per week, and once or more per week. The question on fresh fruit was not comparable to the Oxford WebQ (which asked about consumption of fresh, frozen, and tinned fruit combined) and therefore not used. To rank the women by total fruit intake (excluding juice) we grouped the intake frequencies across the fruit subtypes, dried fruit, and stewed or tinned fruit and grouped them into the following categories: <5, 6-9, 10-14, 15-19, 20+ pw. We then estimated trends in risk across these baseline categories.

*Milk and ice cream*

We estimated dairy and soya milk intake using the questions on cups of milk intake (including hot cocoa), type of milk consumed, bowls of breakfast cereal, cups of tea, and cups of coffee. Women were asked which types of milk or cream they drink once a week or more often. Those who selected ‘full cream’, ‘semi-skimmed’, or ‘skimmed’ were assigned dairy milk, whereas those who selected ‘soya’ were assigned soya milk. We then estimated their total daily dairy or soya milk consumption by summing their milk intakes assuming the following: 95 mL of milk for each cup of milky drinks, 100 mL of milk for each bowl of breakfast cereal, 35 mL of milk for each cup of tea or coffee. For dairy milk, participants were then divided into fifths and we estimated trends in risk across these baseline categories. For soya milk participants were categorized into low and high and analyses were conducted with high versus low. Ice cream intake was included in the question about the types of milk or cream consumed each week and categorized as high versus low.

*Wholegrains*

We estimated wholegrain intakes using questions on intakes of pasta, rice, brown/wholemeal bread, crackers/crispbread, sweet biscuits, cake/puddings, bran cereal, biscuit cereal, oat cereal, muesli, and other cereal types. We used the wholegrain content of foods consumed in the UK to calculate the grams of wholegrains in each item [1] and then totalled the gram intakes across these foods to get a total intake for each woman. Participants were then divided into fifths of intakes and we estimated trends in risk across these baseline categories. The mean intake of 42.8 g of wholegrains in the highest quintile is equivalent to the amount in 2.5 slices of wholemeal bread.

*Remaining foods and beverages*

Women were asked to report how often they consumed the remaining foods and beverages which we categorised as follows based on the distribution of the data: chips (0, 1, 2+ times pw), potatoes <3, 3, 4, 5, 6+ times pw), pasta/spaghetti (0, 1, 2+ times pw), rice (0, 1, 2+ times pw), cheese (<1, 2, 3, 4+ times pw), eggs (<1, 2, 3, 4+ whole eggs pw), slices/pieces of white bread (0, 1-9, 10-19, 20+ slices pw), slices/pieces of wholemeal bread (0, 1-9, 10-19, 20+ slices pw), crackers/crispbreads (0,1-6,7+ number pw), sweet biscuits (0, 1-9, 10+ number pw), dairy desserts (<2, 3-4, 5-6, 7+ number pw), cakes/puddings/pies/buns (0, 1-2, 3+ number pw), chocolate (<1, 2-3, 4-5, 6+ pieces pw), nuts (<1, 1, 2+ Tbs pw), soup (0, 1, 2, 3+ bowls pw), gravy/cream cheese/sauces (<1, 2-3, 4-5, 6+ Tbs pw), crisps (0, 1, 2, 3+ packets pw), boiled sweets(0, 1, 2-6, 7+ number pw), jam (0, 1, 2, 3+ Tbs pw), breakfast cereal (<7, 7, 8+ bowlspw), alcohol (0, 1-5, 6-10, 11+ drinks pw), tea (0-1, 2-3, 4-5, 6+ cups pd), coffee (0-1, 2-3, 4-5, 6+ cups pd), water, fizzy/soft drinks (0, 1, 2+ glasses pd), fruit juice (<2, 2-4, 5-7, 8+ glasses pw), fruit squash (0, 1, 2+ glasses pd), and ice cream (<1, 1+ (portion assumed) pw). We then estimated trends in risk across these baseline categories.

*Nutrients*

We grouped women into fifths and then estimated trends in risk across these five baseline categories.

## Measurement error correction

In this example for calcium, we show the mean intakes in the baseline dietary survey and in the first Oxford WebQ women completed by quintiles of calcium intake in the baseline dietary survey. We assigned the mean Oxford WebQ intakes in women who completed at least one Oxford WebQ to each baseline quintile for all women, and calculated a trend variable.

| Supplementary Table 1. Relative risks and 95% confidence intervals of colorectal cancer per 300 mg/day higher intake of calcium based on calcium intakes from the baseline dietary survey and based on calcium intakes from the baseline dietary survey and re-measured calcium intakes from the Oxford WebQ combined | | |
| --- | --- | --- |
| Baseline dietary survey (N=542,778) | | Oxford WebQ (N=36,597) |
| Categories of calcium intake | Mean calcium intake (mg/day) | Mean calcium intake (mg/day) |
| Q1 | 492 | 828 |
| Q2 | 688 | 912 |
| Q3 | 818 | 970 |
| Q4 | 959 | 1037 |
| Q5 | 1252 | 1126 |
| *Range (mg/d) Q1 to Q5* | *760* | *298* |
| RR^1^, 95% confidence intervals per 300 mg | 0.94, 0.92-0.97^2^ | 0.83, 0.77-0.89^3^ |

^1^Associations between calcium and colorectal cancer incidence separately using Cox proportional hazards regression models that were stratified by year of birth, date of completion of the dietary survey (which is the baseline for this study), and region of residence (10 geographical regions: 9 in England and 1 in Scotland), and adjusted for socioeconomic group (fifths, based on the Townsend deprivation score, unknown), highest educational qualification (none, technical, secondary, tertiary, unknown), body mass index (<20, 20-22.49, 22.5-24.9, 25.0-27.49, 27.5-29.9, 30-32.49, 32.5-34.9, 35+ kg/m2, unknown), height (<160, 160–164.9, ≥165 cm, unknown), strenuous exercise (none, ≤ once per week, > once per week, unknown), dietary energy intake (except for the analysis of energy and risk; fifths, unknown), alcohol (none, 1-5, 6-10, ≥ 11 drinks per week, unknown), smoking (never, past, current 1–4, current 5–9, current <10, current 10–14, current 15–19, current 20–24, current 25–29, current ≥30 cigarettes per day, unknown), current use of hormonal therapy for menopause (no, yes, unknown), and family history of bowel cancer (no, yes). ^2^ Trend uses measured intakes from the baseline dietary survey ^3^ Trend uses the five re-measured intakes from the Oxford WebQ.

# Supplementary Figures


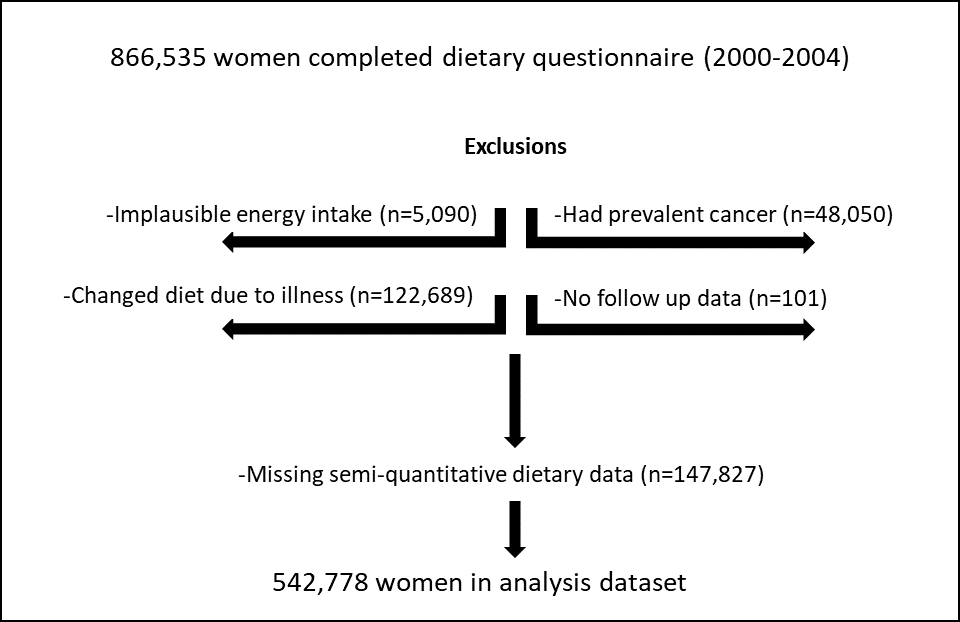


# Supplementary Figure 1. Participant flowchart

# References

1. Jones, A.R., K.D. Mann, S.A. Kuznesof, D.P. Richardson, and C.J. Seal, *The whole grain content of foods consumed in the UK.* Food Chem, 2017. **214**: p. 453-459.
