# Supplementary material for: Diet-wide analyses for risk of colorectal cancer: prospective study of 12,251 incident cases among 542,778 women in the UK
Source: Nat Commun. 2025 Jan 8;16:375. doi: 10.1038/s41467-024-55219-5 (PMC11711514; doi:10.1038/s41467-024-55219-5)
Supplement: Supplementary file 2 — Description of Additional Supplementary Files [file 41467_2024_55219_MOESM2_ESM.docx]

**Supplementary Data 1. Relative risks and 95% confidence intervals for the associations of 97 dietary factors with risk of colorectal cancer, sorted by p values**

^1^Associations between each of the 97 foods or nutrients and colorectal cancer incidence calculated separately using Cox proportional hazards regression models that were stratified by year of birth, date of completion of the dietary survey (which is the baseline for this study), and region of residence (10 geographical regions: 9 in England and 1 in Scotland), and adjusted for area-based deprivation (fifths, based on the Townsend deprivation score, unknown), highest educational qualification (none, technical, secondary, tertiary, unknown), body mass index (<20, 20-22.49, 22.5-24.9, 25.0-27.49, 27.5-29.9, 30-32.49, 32.5-34.9, 35+ kg/m2, unknown), height (<160, 160–164.9, ≥165 cm, unknown), strenuous exercise (none, ≤ once per week, > once per week, unknown), dietary energy intake (except for the analysis of energy and risk; fifths, unknown), alcohol (none, 1-5, 6-10, ≥ 11 drinks per week, unknown), smoking (never, past, current 1–4, current 5–9, current <10, current 10–14, current 15–19, current 20–24, current 25–29, current ≥30 cigarettes per day, unknown), current use of hormonal therapy for menopause (no, yes, unknown), and family history of bowel cancer (no, yes). For each of the 62 quantitatively measured dietary factors, we created a continuous variable using the re-measured mean intakes for each baseline category. Log-linear trends in risk across categories of baseline intakes were then calculated using the listed increments.

**Supplementary Data 2. Associations of FDR-significant dietary factors with risk of colorectal cancer by level of adjustment for lifestyle factors**

Model 1: associations between each of the 17 foods or nutrients and colorectal cancer incidence calculated separately using Cox proportional hazards regression models that were stratified by year of birth, date of completion of the dietary survey (which is the baseline for this study), and region of residence (10 geographical regions: 9 in England and 1 in Scotland); Model 2: associations additionally adjusted for area-based deprivation (fifths, based on the Townsend deprivation score, unknown), highest educational qualification (none, technical, secondary, tertiary, unknown), body mass index (<20, 20-22.49, 22.5-24.9, 25.0-27.49, 27.5-29.9, 30-32.49, 32.5-34.9, 35+ kg/m2, unknown), height (<160, 160–164.9, ≥165 cm, unknown), strenuous exercise (none, ≤ once per week, > once per week, unknown), smoking (never, past, current 1–4, current 5–9, current <10, current 10–14, current 15–19, current 20–24, current 25–29, current ≥30 cigarettes per day, unknown), current use of hormonal therapy for menopause (no, yes, unknown), and family history of bowel cancer (no, yes); Fully adjusted RR: associations additionally adjusted for dietary energy intake (except for the analysis of energy and risk; fifths, unknown), alcohol (none, 1-5, 6-10, ≥ 11 drinks per week, unknown).

**Supplementary Data 3. Associations of FDR-significant dietary factors and colorectal cancer risk by self-reported health and follow-up period**

^1^Associations between each of the 17 foods or nutrients and colorectal cancer incidence calculated separately using Cox proportional hazards regression models that were stratified by year of birth, date of completion of the dietary survey (which is the baseline for this study), and region of residence (10 geographical regions: 9 in England and 1 in Scotland), and adjusted for area-based deprivation (fifths, based on the Townsend deprivation score, unknown), highest educational qualification (none, technical, secondary, tertiary, unknown), body mass index (<20, 20-22.49, 22.5-24.9, 25.0-27.49, 27.5-29.9, 30-32.49, 32.5-34.9, 35+ kg/m2, unknown), height (<160, 160–164.9, ≥165 cm, unknown), strenuous exercise (none, ≤ once per week, > once per week, unknown), dietary energy intake (except for the analysis of energy and risk; fifths, unknown), alcohol (none, 1-5, 6-10, ≥ 11 drinks per week, unknown), smoking (never, past, current 1–4, current 5–9, current <10, current 10–14, current 15–19, current 20–24, current 25–29, current ≥30 cigarettes per day, unknown), current use of hormonal therapy for menopause (no, yes, unknown), and family history of bowel cancer (no, yes).

**Supplementary Data 4. Associations of FDR-significant dietary factors and colorectal cancer risk by cancer site**

^1^Associations between each of the 17 foods or nutrients and colorectal cancer incidence calculated separately using Cox proportional hazards regression models that were stratified by year of birth, date of completion of the dietary survey (which is the baseline for this study), and region of residence (10 geographical regions: 9 in England and 1 in Scotland), and adjusted for area-based deprivation (fifths, based on the Townsend deprivation score, unknown), highest educational qualification (none, technical, secondary, tertiary, unknown), body mass index (<20, 20-22.49, 22.5-24.9, 25.0-27.49, 27.5-29.9, 30-32.49, 32.5-34.9, 35+ kg/m2, unknown), height (<160, 160–164.9, ≥165 cm, unknown), strenuous exercise (none, ≤ once per week, > once per week, unknown), dietary energy intake (except for the analysis of energy and risk; fifths, unknown), alcohol (none, 1-5, 6-10, ≥ 11 drinks per week, unknown), smoking (never, past, current <10, current 10–14, current 15–19, current 20–24, current 25–29, current ≥30 cigarettes per day, unknown), current use of hormonal therapy for menopause (no, yes, unknown), and family history of bowel cancer (no, yes). Phet stands for p for heterogeneity.

**Supplementary Data 5. Relative risks^1^ and 95% confidence intervals for the association of the FDR-significant dietary factors and risk of colorectal cancer stratified by lifestyle factors (excluding alcohol)**

^1^Associations between each of the 17 foods or nutrients and colorectal cancer incidence calculated separately using Cox proportional hazards regression models that were stratified by year of birth, date of completion of the dietary survey (which is the baseline for this study), and region of residence (10 geographical regions: 9 in England and 1 in Scotland), and adjusted for area-based deprivation (fifths, based on the Townsend deprivation score, unknown), highest educational qualification (none, technical, secondary, tertiary, unknown), body mass index (<20, 20-22.49, 22.5-24.9, 25.0-27.49, 27.5-29.9, 30-32.49, 32.5-34.9, 35+ kg/m2, unknown), height (<160, 160–164.9, ≥165 cm, unknown), strenuous exercise (none, ≤ once per week, > once per week, unknown), dietary energy intake (except for the analysis of energy and risk; fifths, unknown), alcohol (none, 1-5, 6-10, ≥ 11 drinks per week, unknown), smoking (never, past, current 1–4, current 5–9, current <10, current 10–14, current 15–19, current 20–24, current 25–29, current ≥30 cigarettes per day, unknown), and family history of bowel cancer (no, yes). Phet stands for p for heterogeneity.

**Supplementary Data 6. Average food and nutrient intakes in first Oxford WebQ (N=36,597)**
