# Supplementary material for: Diet-wide analyses for risk of colorectal cancer: prospective study of 12,251 incident cases among 542,778 women in the UK
Source: Nat Commun. 2025 Jan 8;16:375. doi: 10.1038/s41467-024-55219-5 (PMC11711514; doi:10.1038/s41467-024-55219-5)
Supplement: Supplementary file 4 — Reporting Summary [file 41467_2024_55219_MOESM4_ESM.pdf]

nature portfolio

Corresponding author(s): Keren Papier

Last updated by author(s): Nov 5, 2024

Reporting Summary

Nature Portfolio wishes to improve the reproducibility of the work that we publish. This form provides structure for consistency and transparency in reporting. For further information on Nature Portfolio policies, see our Editorial Policies and the Editorial Policy Checklist.

Statistics

For all statistical analyses, confirm that the following items are present in the figure legend, table legend, main text, or Methods section.

n/a	Confirmed	
	x	The exact sample size (*n*) for each experimental group/condition, given as a discrete number and unit of measurement
	x	A statement on whether measurements were taken from distinct samples or whether the same sample was measured repeatedly
	x	The statistical test(s) used AND whether they are one- or two-sided<br>*Only common tests should be described solely by name; describe more complex techniques in the Methods section.*
	x	A description of all covariates tested
	x	A description of any assumptions or corrections, such as tests of normality and adjustment for multiple comparisons
	x	A full description of the statistical parameters including central tendency (e.g. means) or other basic estimates (e.g. regression coefficient) AND variation (e.g. standard deviation) or associated estimates of uncertainty (e.g. confidence intervals)
	x	For null hypothesis testing, the test statistic (e.g. *F*, *t*, *r*) with confidence intervals, effect sizes, degrees of freedom and *P* value noted<br>*Give P values as exact values whenever suitable.*
x		For Bayesian analysis, information on the choice of priors and Markov chain Monte Carlo settings
x		For hierarchical and complex designs, identification of the appropriate level for tests and full reporting of outcomes
	x	Estimates of effect sizes (e.g. Cohen's *d*, Pearson's *r*), indicating how they were calculated

Our web collection on statistics for biologists contains articles on many of the points above.

Software and code

Policy information about availability of computer code

Data collection	Information on data access is available at www.millionwomenstudy.org/data_access/.GECCO collaborators and consortium members can access the GECCO portal here: https://research.fredhutch.org/peters/en/genetics-and-epidemiology-of-colorectal-cancer-consortium.html. The code for the MR analysis in this study can be found: https://github.com/karlsmithbyrne/Lactase_MR/tree/main.
Data analysis	Stata version 18.1 , and R version 4.1.2 were used to conduct all statistical analyses.

For manuscripts utilizing custom algorithms or software that are central to the research but not yet described in published literature, software must be made available to editors and reviewers. We strongly encourage code deposition in a community repository (e.g. GitHub). See the Nature Portfolio guidelines for submitting code & software for further information.

Data

Policy information about availability of data

All manuscripts must include a data availability statement. This statement should provide the following information, where applicable:

- Accession codes, unique identifiers, or web links for publicly available datasets
- A description of any restrictions on data availability
- For clinical datasets or third party data, please ensure that the statement adheres to our policy

Provide your data availability statement here.

Research involving human participants, their data, or biological material

Policy information about studies with human participants or human data. See also policy information about sex, gender (identity/presentation), and sexual orientation and race, ethnicity and racism.

Reporting on sex and gender	This work reported on sex.
Reporting on race, ethnicity, or other socially relevant groupings	In the MR analysis only genetically predicted Europeans were considered.
Population characteristics	The study is based on data from 542,778 women with a mean (SD) age of 56 (6) years.
Recruitment	The Million Women study recruited 1.3 million women between 1996-2001 who were invited to the National Health Service (NHS) Breast Screening Programme in England and Scotland joined the Million Women Study by completing the recruitment questionnaire.
Ethics oversight	The study was approved by the Oxford and Anglia Multi-Centre Research Ethics Committee and all participants gave written consent for follow-up through medical records.

Note that full information on the approval of the study protocol must also be provided in the manuscript.

Field-specific reporting

Please select the one below that is the best fit for your research. If you are not sure, read the appropriate sections before making your selection.

- [x] Life sciences
- [ ] Behavioural & social sciences
- [ ] Ecological, evolutionary & environmental sciences

For a reference copy of the document with all sections, see nature.com/documents/nr-reporting-summary-flat.pdf

Life sciences study design

All studies must disclose on these points even when the disclosure is negative.

Sample size	The maximal analysis cohort included 542,778 participants, the genetic analysis contained up to 99,152 participants.
Data exclusions	In total 866,535 women completed the baseline dietary questionnaire and had linked data for cancer and death. Of these, we excluded: 48,151 women with previous registration for malignant cancer (other than non-melanoma skin cancer, C44) or no follow-up before the dietary questionnaire completion date; 5,090 women whose energy intake was outside the range 2093-14,654 KJ per day; 122,689 women who reported having changed their diet due to illness; and 147,827 women with missing data on any semi-quantitative dietary variables (including meat types, fish types, main carbohydrate sources, eggs, vegetables, fruit, sweets, dairy, alcohol, and other beverages) leaving 542,778 women in the final analysis dataset.
Replication	Replication will be required on an external dataset.
Randomization	Not relevant because this is an observational cohort study
Blinding	Not relevant because this is an observational cohort study

Reporting for specific materials, systems and methods

We require information from authors about some types of materials, experimental systems and methods used in many studies. Here, indicate whether each material, system or method listed is relevant to your study. If you are not sure if a list item applies to your research, read the appropriate section before selecting a response.

Materials & experimental systems

n/a	Involved in the study	
x		Antibodies
x		Eukaryotic cell lines
x		Palaeontology and archaeology
x		Animals and other organisms
x		Clinical data
x		Dual use research of concern
x		Plants

Methods

n/a	Involved in the study	
x		ChIP-seq
x		Flow cytometry
x		MRI-based neuroimaging

Plants

Seed stocks	NA
Novel plant genotypes	NA
Authentication	NA
